# On Yellow and Break-Bone Fevers

**Published:** 1881-01

**Authors:** J. C. Le Hardy


ARTICLE II.

### ON YELLOW AND BREAK-BONE FEVERS.

BY PROF. J. C. LE HARDY, M. D.

SAVANNAH, *November 20th, 1880.*

MR. EDITOR.—Having just passed through the ordeal of a wide-spread epidemic of break-bone (Dengue) fever, it has occurred to me that a simple and unvarnished description of this disease, written while its features are still fresh in my memory, might prove interesting to your readers; and as some writers have laid down the doctrine that yellow fever and break-bone fever are one and the same, that dengue is only a mild form of the dreaded *vomito;* I have taken this occasion to place the symptoms of the two diseases before the profession in such close juxta position, that every one may be able to form his own judgment upon this point.

Break-bone fever (the best name *yet found* for this ill) like yellow fever, belongs to the intensely malarial zone following the coast and river courses below the 34th parallel of north latitude. Like it, it returns at indefinite and irregular periods in the epidemic form; the epidemic being at times localized at others spread over considerable extent of country.

In both diseases the occurrence of an epidemic is preceded and attended by special conditions of the surrounding media—both earth and air. These conditions are so nearly alike that both diseases may exist at the same time in the same locality, or one may give place for a time to the other and become again the prevailing disease. These circumstances occurred in this city both in 1854 and in 1858, as you may remember. Like the winters preceding the yellow fever epidemics of 1817, 1820, 1854 and 1876. Our last winter was very mild, the frost was, indeed, so slight that the most tender plants in our gardens did not suffer. Our people, therefore, felt some apprehension as to the summer which was to follow. But the spring and the early part of summer were very dry in contrast with that of the above-named years. The dryness of the soil in this section was, indeed, something remarkable; our deepest swamps became perfectly dry, and so remained, and our malarial fevers did not appear as usual in May, or June, or July, or even the first half of August. Doctors had nothing to do, their faces were much elongated.

In 1876 rain poured in large quantities in June and July, while in 1880 the wet season only started in August. In 1876 the heat became oppressive in June; in 1880 the latter part of July, continuing in August. Malarial fevers were numerous in June, and their type increased in intensity in July, and by the middle of August in 1876 yellow fever became epidemic.

In 1880, on the contrary, cases of malarial fever were of rare occurrence until after the fall of heavy rains in August, and these soon changed their type into that of break-bone. Our epidemic starting about the 27th of the month, cases being reported in various parts of the town at that date. In a short time it had invaded almost every house in the city and its suburbs.

The break-bone epidemic atmosphere of 1880, like the yellow fever atmosphere of 1878 extended over a vast area of country. It raged in a great number of cities and villages south of the 34th parallel. Cases even occurred in the most isolated spots in our pine forests.

The symptoms of break-bone, like those of yellow, or any other fevers, occurring in the epidemic form, are extremely varied. In our epidemic they were in some cases so slight as hardly to produce any discomfort, the only objective symptom being the appearance of the characteristic eruption about the wrists. In others, although the temperature remained normal, the other symptoms, of intense pain in various parts of the body, gastric irritation, and vomiting and great prostration were present and in bold relief. In others again the temperature would rise so high and remain up for so long a period, the vomiting would be so persistent, the pains so excruciating and the resulting prostration so alarming that it might well be wondered how the patient could pass through such an ordeal and yet recover.

Thinking it would not be practicable or even desirable to describe, in an article of the scope of this one, the different shades of intensity in which this fever appears ; I have simply selected from my book of records a case in which the main features of the disease were well marked, giving thus to your readers, a bedside view of the break-bone fever, and have followed the same method to bring before their mind's eye a severe but not fatal case of yellow fever, and a violent and fatal case of the same disease, in order to make the following table of comparison easily understood :

### Typical Case of Break-Bone Fever.

Mrs. E. P——, age 28; Irish; Temperament sanguine; full habit. September 4, 1880.—Had a chill 7.30 A. M.; examined at 8.30 A. M. Decubitus dorsal, complains of chilly sensations up and down the back; face flushed; eyes suffused and watery, sensitive to light; skin very hot to the touch; frontal headache, pains in the back and limbs, very severe; Temperature 104.5, Pulse 132, Respiration 40; tongue coated on each side; centre clean; tip red.

4 P. M.—T. 105, R. 40, P. 128; vomiting green and yellow bile and remnants of food; vomit very acid.

8 P. M.—T. 105.5, R. 44, P. 124; vomiting frequent; bile and mucus thrown up; very acid; pains excruciating in head, back and limbs; skin very sensitive, painful to the touch; tongue entirely coated; tip red.

September 5, 9 A. M.—T. 103, R. 32, P. 100; spent a sleepless and restless night; still vomiting bile and mucus; acid reaction very strong; urine plentiful; clear, colored with bile, acid sp. gr. 1010; pains severe; color of skin natural; tongue thickly coated, flabby, indented by teeth; tip and edges red; stool bilious; eyes injected, still sensitive to light.

4 P. M.—T. 104, R. 30, P. 100; pains distressing; restless tossing; mind clear; vomits mouthfuls of bile; passed one quart of highly colored but clear urine in last 24 hours; reaction acid; slight cloudiness produced by boiling, on adding nitric acid.

September 6, 9 A. M.—Slept some during preceding night; is quiet; face pale; eyes natural; T. 100, R. 24, P. 84; occasional vomiting of bile and acid fluids; urine highly colored with bile pigments, sp. gr. 1010; tongue red, but moist in centre; tip and edges very red; pains subsiding; tenderness of epigastrium.

4 P. M.—T. 102, R. 24, P. 80, weak; patient feels very feeble; restless, but not tossing; urine highly colored, no more cloudiness on boiling; stool dark brown.

September 7th, 9 A. M.—T. 98, R. 16, P. 64; vomiting ceased; tenderness over epigastrium, liver and spleen; urine scanty, highly colored, acid sp. gr. 1020, deposits water; no motion of bowels; pains almost gone; skin pale; eyes natural in color; an eruption resembling that of measles has appeared on the back of hands and forearms, on the feet and legs; very itchy; patient very much prostrated.

4 P. M.—T. 99.5, R. 14, P. 64; urine copious, clear; acid stool bilious; tongue clearing; eruption spreading about the neck; appetite returning; enjoys beef tea, milk and lime water, champagne, mint julep.

September 8.—T. 98, R. 16, P. 64, weak; great prostration; urine clear, acid; skin pale; eyes languid; eruption fading.

September 9th.—T. 98, R. 16, P. 68; very feeble.

September 10th.—T. 98.5, P. 72; regaining some strength; tongue getting normal, epidermis desquamating. Discharged.

TYPICAL CASE OF YELLOW FEVER TERMINATING IN RECOVERY.

Taken from the record book of epidemic of 1876.

1876, September 14.—B. S——, age 24; clerk; native; sanguine. Had a chill 5 P. M.; visit 6 P. M.; complains of chilly sensations up and down the back; decubitus dorsal; eyes injected, smoky, intolerant of light; face deep red; whole surface of skin congested, heat of skin very pungent to the touch; frontal headache very severe, also pains in back and limbs; restless tossing; sighing repeatedly; tongue coated whitish-yellow; tip red; Temperature 106; Pulse 124; Respiration 48; urine highly colored; acid; no albumen.

September 15, 3 A. M.—T. 106.5, P. 124, R. 52; very restless, tossing about the bed; sighing; pains in head, back and limbs intense.

8 A. M.—T. 104, P. 100, R. 48; urine heavy cloudy; sp. gr. 1015; no albumen; stomach irritable; vomit green, and yellow bile, separated; mucus; particles of food, intensely acid; epigastrium tender on pressure; skin deep red, not sensitive to touch; tongue coated (wash leather); tip red.

5 P. M.—T. 104.5, P. 96, R. 40; restless; tossing; sighing; pains still very severe; muttering delirium, from which he is easily roused; vomits occasionally a clear fluid; mixed ropy mucus. intensely acid; urine shows a deposit on boiling, by the addition of nitric acid, is scanty and thick; bowels constipated; motion by medicine dark brown.

September 16, 8 A. M.—T. 102.5, R. 32, P. 88; eyes still red, turning yellow; circulation of skin much impeded; color deep red, with a yellowish tinge; urine murky, scanty; albumen abundant; neutral; stomach quieter, retains champagne and ice, Seltzer water; pains gone; Tongue dark brown in centre, tip and edges very red; gums spongy and bleeding easily; bleeding from the nasal mucus membrane in the night.

5 P. M.—T. 102, R. 32, P. 88; listless; sleeps a great deal of the time; mind clearer; urine scanty, albuminous; yellowness of skin and conjunctivæ; deepening.

September 17, 8 A. M.—T. 99, R. 20, P. 72; eyes and skin orange yellow; urine freer and clearer, albumen diminishing; patient feels very much prostrated; mind clear; bleeding from nose and gums during the night; tongue brown in centre, red at tip and edges.

5 P. M.—T. 99.5, R. 28, P. 68; stomach quiet, retains beef extract in water, champagne, wine whey; urine much clearer and abundant; slight cloudiness on boiling; skin and eyes of a brighter yellow; progressing favorably, but complains of so much tenderness and pain in epigastrium that a blister was applied.

September 18, 9 A. M.—T. 98, R. 16, P. 64; prostration great; decubitus dorsal; quiet; eyes clearer; epigastric pain relieved; has some desire for food; tongue cleansing: bleeding stopped; stool contains disintegrated black blood; sleep natural; urine clear; no albumen. Discharged.

### Violent Case of Yellow Fever Terminating in Death.

1876, October 5.—J. B——, German; age 24; sympathetic. Had a chill at 6 P. M.; visit 7 P. M.; Pulse 108, Temperature 104, Respiration 44; excruciating pains in head, back and limbs; eyes injected, smoky, ecchimosed in several spots; very restless, tossing about the bed, sighing repeatedly; skin deep red and purplish in spots from capillary stasis; vomited food.

October 6, 7 A. M.—Eyes ecchimosed with a mild expression; P. 92; T. 104, R. 48; skin darker; tossing; sighing; mind clear; urine scanty, muddy, highly albuminous; vomit clear, mixed with mucus, streaked with blood; frontal headache, compared to a bar of hot iron circling forehead.

4 P. M.—T., P. and R. same as at morning visit; vomit clear, with a few brown specks, shreds of mucus membrane, and streaks of blood.

October 7, 8 A. M.—T. 104, P. 68, R. 60; tossing; sighing; vomit clear, very acid, brown specks increasing, contains rolls and shreds of mucus membrane; no urine since last seen, with catheter I removed a tablespoonful, which became almost solid on boiling; eyes reddish yellow, with ecchimosed spots, much darker.

6 P. M.—Symptoms very much as at 8 A. M.; urine suppressed; Temperature 104.5.

October 8,—T. 105, P. 92, R. 60; very restless; expression mild no secretion of urine for last 36 hours; gums spongy and bleeding; tongue brown, parched, cracked; teeth covered with black sordes; skin bronze colored; eyes reddish yellow; patient went into convulsions whilst I was there; whilst in convulsion throws up black vomit in gulps, and with such force that walls, ceilings and attendants were bespattered with it. Death occurred whilst in one of these convulsions.

Post mortem examination made at once. Blood black fluid; liver dark brown; veins filled with black blood; kidneys small, pale; Duodenum filled with disintegrated blood.

### Comparison of Break-Bone and Yellow Fevers.

The two diseases belong to malarial districts in semi-tropical climates, or where the temperature may remain for weeks at from 75° to 85° Fahrn. Both occur in the epidemic form, at irregular intervals, run a definite course, and their epidemicity is always cut short by a frost. Both require a special germ for their production. The poison producing the one seems to have no influence upon the other. Both may be followed by relapses. In their nature they are infectious, but not contagious from persons.

### Yellow Fever.

*General Symptoms.*

Starts with a chill, followed by hot or cold sensations running along the spine; frequently starts in the night. The fever which follows or ex-

### Break-Bone Fever.

*General Symptoms.*

First symptoms, similar to yellow fever, occur at any time of day or night. The fever which exists with or immediately follows the chill is a monoists with the chill is a mono paroxysmal fever, lasting from 60 to 72 hours, accompanied with severe frontal headache, pains in the back and limbs, skin hot, giving an unpleasant biting sensation to the touch (calor mordax), patient emitting a peculiar mousy smell. The circulation of the skin diminished from congestion of the capillary system, which gives frequently a dark red hue, or a mottled red and purple color to the surface, sensation of the skin diminished.

Temperature ranging from 102 to 107½. The pulse at the onset ranges from 100 to 132, gradually comes down to from 40 to 90. The discrepancy between the pulse and bodily heat well marked in most cases after the second day. Rate of mortality very high.

*Eyes.*

Conjunctiva injected, red or smoky, often ecchymosed in spots, turning yellow on third or fourth day. Photophobia well marked at the onset of the disease.

*Tongue.*

Whitish yellow (wash leather) with red tip, becomes glazed and red in centre, sometimes paroxysmal fever, lasting from 12 to 96 hours, accampanied with severe frontal headache, pains in the limbs, skin gives a hot sensation to the touch, emitting a peculiar mousy smell. The circulation of the skin is not impeded, if any capillary congestion occurs it is evanescent, sensation of surface much increased. Temperature ranges from 100 to 116½. The pulse at the onset ranges from 110 to 140, gradually comes down to from 64 to 86. The discrepancy between bodily heat and pulse well marked in most cases after the second day. Death from the disease of very rare occurrence.

*Eyes.*

Conjunctiva, congested eyes, smoky or watery; returning to their natural color and condition at or near the subsidence of the fever. Photophobia marked at the onset.

*Tongue.*

broad and flabby, with whitish fur and red tip, sometimes red and glazed over whole surface, or in the centre, emitting a very strong and disagreeable odor.

*Gums.*

Firm, often pale. Salivary parched, cracked and brown with sordes on the teeth.

*Gums.*

Spongy, bleeding easily. Salivary glands, secretion, greatly diminished, often entirely suppressed.

*Stomach.*

Occasionally irritable from the first day; vomit intensely acid during the first and second days. Black vomit usually occurring on third or fourth day after subsidence of fever.

*Bowels.*

Usually constipated, passages frequently, bloody, containing black disintegrated blood. No bile present in evacuation after second day.

*Liver.*

Secretions generally suppressed after first day of fever. Liver becomes hard, smaller and turned of a box-wood color (in post mortem sections).

*Spleen.*

Shows no special changes on patients recovering.

*Kidneys.*

Secretion always diminished, often suppressed; albumen present in most of violent cases. Urine thick and muddy. Hemorrhages sometimes occur.

glands, secretion, sometimes diminished, sometimes increased.

*Stomach.*

Generally irritable from first; vomit acid and profuse. Bile vomited during the whole paroxysm of fever. Vomiting subsiding with the fever. No black vomit.

*Bowels.*

Sometimes constipated, sometimes loose. Passages always contain bile; in rare cases bleeding occurs in the bowels; the blood always red.

*Liver.*

Secretions always active during the whole disease. Sometimes becomes enlarged and tender on pressure.

*Spleen.*

Often enlarges and feels tender during and after the subsidence of fever.

*Kidneys.*

Flow of urine remains free, and pretty clear, rarely contains albumen or blood; bleeding very seldom occurs.

*Skin.*

Skin slightly congested at first, shows little change of color throughout, generally paler at termination of fever. If yel-

*Skin*

Congested, red or mottled from the onset, circulation often much impeded. Turns yellow and deep orange color on third or 4th day. Sensibility of skin diminished. Yellowness of skin persisting for a long time. Marked tendency to purpura hemorrhagica. Eruption seldom occurs and comes on body and chest.

*Blood.*

A change perceptible to the senses occurs on the second or third day, it becomes much darker, less coagulable, and may become unfit for carrying on the functions of life on the second or third day.

*Nervous System.*

Becomes deeply involved, especially the great sympathetic controlling secretions, which, after the first day of the fever, seems to be paralyzed in its functions; general sensation also diminished with the progress of the fever. Debility following the attack very great.

*The Cause.*

In my opinion it is produced by the inhalation of a poison contained in the spores of a fungus floating in the air, which, lowish, from a check in the flow of bile, this discoloration disappears in a day or two. Sensitiveness of skin intensified. Purpura very rare. Eruption very frequent occurring, principally on extremities.

*Blood.*

No perceptible change takes place in the blood, it remains bright red and coagulable. White cells increased after third or fourth day.

*Nervous System.*

General sensation is increased. The nerves controlling secretion remain active throughout the progress of the disease. Debility very great and lasting.

*The Cause.*

I believe it to be produced by the inhalation and absorption of the spores of a different species of fungus, the effects of if absorbed in large quantity, will produce such chemical changes in the blood as to paralyze the ganglionic system of nerves, and death takes place from inaction of the glands, the blood being unable to rid itself of the poison and of its own effete material. which are to produce such a chemical change in the blood as to stimulate instead of paralyzing both the sensory and the garglionic nervous systems, and that this poison is entirely eliminated by the glands.

